# Generation of an endogenous auxin inducible degron-tagged SPAS-1/spastin to investigate its targeted depletion in *C. elegans* neurons

**DOI:** 10.17912/micropub.biology.001328

**Published:** 2024-11-07

**Authors:** Emily Brown, Samantha Kuszynski, Faith Akoachere, James Feduccia, Lili Malatinszky, Eric S. Luth

**Affiliations:** 1 Department of Biology, Simmons University, Boston, Massachusetts, United States

## Abstract

To facilitate investigations of the microtubule severing protein spastin and its specific role in neurons, we aimed to create a *C. elegans* strain in which the spastin homolog SPAS-1 is visible and can be degraded with spatial and temporal precision. We used CRISPR-Cas9 to fuse an auxin-inducible degron and mScarlet to the endogenous SPAS-1 protein, enabling degradation of SPAS-1 in neurons during desired life stages. DNA sequencing confirmed in-frame insertion with the SPAS-1 N-terminus and fluorescence microscopy revealed endogenous SPAS-1 throughout the CRISPR-edited worms. Auxin treatment in rgef-1::TIR1; mScarlet::AID*::3xFLAG::spas-1 animals reduced mScarlet::SPAS-1 fluorescence in neuronal ganglia.

**Figure 1. Tagging and degradation of endogenous SPAS-1:**
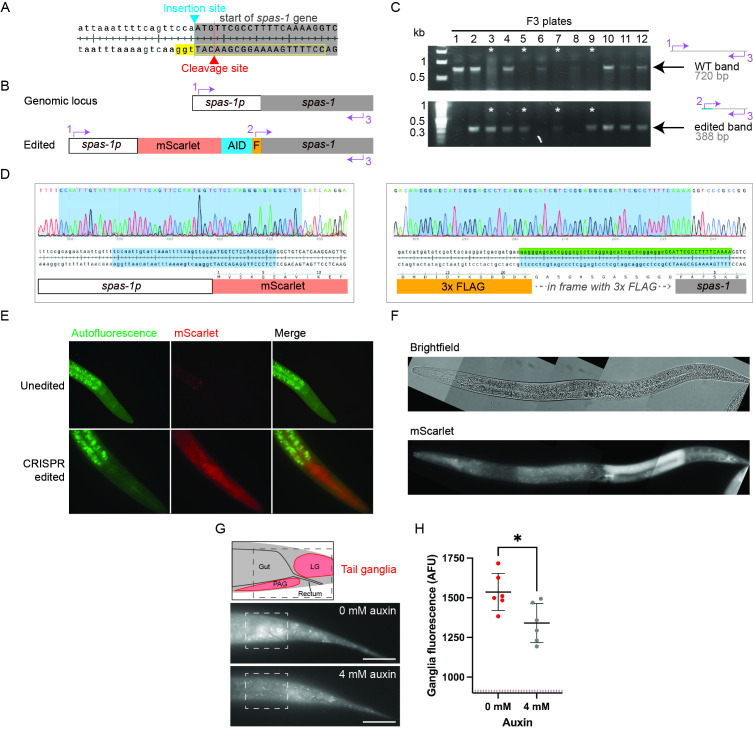
A) Details of the Cas9 cleavage site (red triangle) and tag insertion site (blue triangle) at the *spas-1* locus. The desired insertion site was immediately upstream of the *spas-1* start ATG on the sense strand. The 5'-3' “tgg” on the antisense strand (highlighted in yellow) was used as a PAM sequence, and the 20 nucleotides in the 5' direction (boxed in yellow) were used as the gRNA targeting sequence. B) Diagram of the unedited and edited *spas-1* locus showing the insertion of mScarlet, AID, and the 3x FLAG tag plus linker (noted by “F”). Positions of the primers used in panel C are indicated by numbered purple arrows. C) PCR detection of wildtype and edited *spas-1* in F3 worms following injection of P0 adults. Top shows the “WT” 720 bp product amplified from unedited DNA using primers 1 and 2. Bottom shows the “edited” 388 bp product using primers 2 and 3. * = plates with worms that are homozygous for the CRISPR edit. D) DNA sequencing of the 5' junction between the *spas-1* promoter and start of the mScarlet sequence (left) and the 3' junction between the FLAG-linker sequence and the start of the *spas-1* gene (right). Sequencing chromatograms (shown above) match predicted sequences (shown below). E) A comparison of mScarlet fluorescence in the heads of unedited (top) and edited (bottom) worms. F) Representative composite brightfield and mScarlet images of an L4 animal. G) Locations of the preanal ganglion (PAG) and lumbar ganglion (LG) of the *C. elegans* tail and mScarlet fluorescence in the tails of edited worms in the absence and presence of 4 mM auxin for 24 hours. Dashed box in the top diagram corresponds to the boxed region in the images below. Scale bar = 20 μm. H) Mean fluorescence of the boxed regions shown in E with and without auxin. Gray and red dashed lines along the bottom of the graph correspond to the average background fluorescence levels with and without auxin, respectively. * p<0.05 unpaired t-test.

## Description

Mutations in the gene encoding spastin, a cytoskeleton-associated protein, cause hereditary spastic paraplegia (Liu et al., 2021) . This condition is associated with the degeneration of corticospinal axons due to the breakdown of membrane compartments and the cytoskeletal network (Liu et al., 2021) . *C. elegans* have a spastin homolog called SPAS-1 (Frohlich, 2001, Matsushita-Ishiodori et al., 2007), which, like spastin, displays an ATP-dependent microtubule severing function (Errico et al., 2007, Salinas et al., 2005, Matsushita-Ishiodori et al., 2007) . In *C. elegans* the regulation of microtubules by SPAS-1 is important for synapse remodeling (Kurup et al., 2015) , but SPAS-1 is expressed in tissues other than neurons, such as the intestine (Brown and Bejjani, 2017) . In order to facilitate studies in *C. elegans* that explore the regulation of spastin/ SPAS-1 and its specific role in neurons, we set out to create a worm strain in which the endogenous SPAS-1 protein is visible and able to be degraded with spatial and temporal precision. To do so, we used CRISPR/Cas9 gene editing in conjunction with auxin-inducible degradation (AID). AID relies on the co-expression of the E3 ubiquitin ligase TIR1 in desired cells (spatial control) and the administration of exogenous auxin (temporal control) to provide rapid degradation of a protein of interest (Nishimura et al., 2009, Ashley et al., 2021) .

To avoid interfering with the microtubule-severing C-terminus of SPAS-1 (Onitake et al., 2012) , we elected to target the N-terminus for our CRISPR edit, utilizing the PAM site at the *spas-1* start codon ( Fig. 1A ). We injected Cas9 ribonucleoproteins and a repair template consisting of mScarlet::AID::3xFLAG::linker ( Fig. 1B ) (Ashley et al., 2021) into DV3805 worms that express the necessary AID enzyme TIR1 specifically in neurons (Ashley et al., 2021) . After microinjection, we performed PCR with two sets of primers to distinguish between CRISPR-edited and unedited offspring: Primers 1 and 3 were used to produce a 720 bp amplicon in the unedited *spas-1* locus. Since Primer 2 binds to the FLAG::linker sequence of our repair template, it was used in conjunction with Primer 3 to produce a 388 bp amplicon that confirms the presence of our insertion ( Fig. 1B ). Lysis and PCR of F3 worms revealed plates that did not yield the 720 bp unedited amplicon ( Fig. 1C top) but did yield an amplicon with the edit-specific primer ( Fig. 1C bottom), suggesting that they were homozygous for the CRISPR edit. Subsequent PCR of homozygous knock-in animals with Primers 1 and 3 using a longer extension time revealed the expected ~1.9 kb knock-in specific band. To confirm that our edit was in frame with *spas-1* , we sequenced the junctions between our repair template and the genomic locus. Sequencing revealed an in-frame alignment of the tag with genomic *spas-1* ( Fig. 1D ), suggesting that our edited worms should produce mScarlet::AID::SPAS-1, and fluorescence microscopy confirmed that the mScarlet tag was functional ( Fig. 1E ). We were able to detect mScarlet::AID::SPAS-1 fluorescence throughout L4 animals including in the gonad and vulva ( Fig. 1F ).

To validate our strain, we compared fluorescence levels in worms treated with and without auxin, focusing on a region in the tail where the fluorescence of neuronal ganglia is easily observed ( Fig. 1G ). Auxin treatment resulted in a statistically significant reduction in mScarlet fluorescence in this region ( Fig. 1H ) while other fluorescence is still visible, suggesting that we can precisely degrade endogenous SPAS-1 in neurons. This new strain can be used to identify and characterize regulators of the expression, localization, dynamics, and degradation of endogenous SPAS-1 . It can also be used to precisely assess the role of neuronal SPAS-1 across different developmental stages or crossed with other TIR1 transgenes to degrade SPAS-1 in other tissues.

## Methods

*C. elegans* strains were housed at 20 °C and maintained according to standard procedures (Brenner 1974) . mScarlet::AID*::3xFLAG::linker was amplified from pJW2098 (a gift from Jordan Ward [Addgene plasmid # 163094]) (Ashley et al., 2021) using 5' SP9 modified-primers with and, separately, without 35 nt homology arms flanking the insertion site at the *spas-1* genomic locus. Melting and reannealing a mixture of repair templates with and without homology arms produces mixed strands with asymmetric, single stranded homology arms that are better able to be incorporated via homology-directed insertion (Dokshin et al. 2018) . Primers were as follows with homology arms in parentheses, FWD: (aattgttttccaattgtattaaattttcagttcca)ATGGTCTCCAAGGGAGAGGC, REV: (GTTGAGCTGCCGGCGGGACCTTTTGAAAAGGCGAATCC)gcctccggacgatgctcctg. Amplified template was purified using Mag-Bind TotalPure NGS magnetic beads (Omega Biotek) as described (Ghanta et al., 2021) . Cas9/gRNA ribonucleoprotein complexes (RNPs) containing the universal Alt-R CRISPR-Cas9 tracrRNA (IDT), custom Alt-R CRISPR-Cas9 crRNA (locus-specific sequence CCUUUUGAAAAGGCGAACAU) (IDT), and *S. pyogenes* Cas9-NLS protein (QB3 MacroLab) were assembled as described by Ghanta et al. (2021). Purified repair template was melted according to Ghanta and Mello (2020) and combined with assembled RNPs, *pRF4* ( *rol-6 ( su1006 ))* injection marker (Addgene 92364) at a final concentration of 40 ng/μl (Mello et al., 1991) , and nuclease free water to form a 20 μl injection mix. Adult DV3805 worms (Ashley et al., 2021) were injected and F1 offspring selected as described (Ghanta et al., 2021) . Auxin treatment was performed for 24 hours on nematode growth medium plates with 4 mM synthetic auxin (1-naphthaleneacetic acid) (Sigma 317918) as described (Martinez et al., 2020) .

For fluorescence microscopy, worms were anesthetized in 30 mg/ml 2,3-Butanedione monoxime (Sigma B0753) in M9 buffer (KH _2_ PO _4_ , 22.0 mM; Na _2_ HPO _4_ , 42.3 mM; NaCl, 85.6 mM autoclaved, plus 1 mM sterile MgSO _4_ ) and mounted on 2% agarose pads. Worms were imaged with a 63x objective on an Axio Observer (Carl Zeiss) microscope equipped with an Orca-4 camera (Hamamatsu) and illuminated with a Colibri 7 LED. For autofluorescence images, worms were exposed for 150 ms with 80% LED intensity and 450-490 nm excitation, 500-550 nm emission filters. For mScarlet images, worms were exposed for 1 s with 80% LED intensity and 574-599 nm excitation, 612-682 nm emission filters. Tail fluorescence quantification was performed using FIJI (Schindelin et al., 2012) . A 20 x 30 μm region of interest (ROI) was drawn around the tail ganglia with the edge of the rectum placed in the lower corner. To subtract the background fluorescence, automatic thresholding was applied using the Minimum setting. The average minimum ROI fluorescence in the box (background fluorescence) was used as a floor for the X axis in the resulting graph of mean ROI fluorescence. Statistical analysis was performed using GraphPad Prism version 10.

## Reagents

**Table:** 

**Plasmid**	**Genotype**	**Description**
pJW2098	DH5alpha with insert: 30aa linker:: mScarlet(dpi)::AID*::3xFLAG::10aa linker	Source of tag used for CRISPR repair template. Available from Addgene.
pRF4	DH5alpha with insert: 4kb EcoRI fragment of *C.elegans* genomic DNA containing *rol-6 ( su1006 ) *	Dominant negative roller co-injection marker. Available from Addgene.

**Table:** 

**Strain**	**Genotype**	**Available From**
DV3805	reSi7 [rgef-1p::TIR1::F2A::mTagBFP2::AID*::NLS::tbb-2 3'UTR ] *I*	CGC
ESL27	*reSi7 I;* spas-1 ( sim1 [mScarlet::AID*::3xFLAG::spas-1]) V	Luth lab
